# Concluding remarks: *Faraday Discussion* on Frontiers in physical chemistry in lignin valorization

**DOI:** 10.1039/d5fd00153f

**Published:** 2025-12-10

**Authors:** Katalin Barta

**Affiliations:** a Institute of Chemistry, University of Graz Austria katalin.barta@uni-graz.at

## Abstract

The valorisation of lignin, the only high-volume, naturally occurring aromatic biopolymer, represents an essential frontier for the sustainable production of chemicals and materials. However, despite its tremendous potential, lignin still remains the “dark horse” of biorefining – harbouring great promise yet still insufficiently understood. This closing personal perspective summarizes the recent *Faraday Discussion* on ‘Frontiers in physical chemistry for lignin valorisation’, framing the multidisciplinary efforts required to transition this field into industrial viability. The meeting presented work across four critical domains: fundamental structural understanding, stabilization during extraction and processing, conversion of technical lignins, and strategies for compatibilisation with commercial applications. The discussion reinforced that future success hinges on achieving mastery over several interconnected domains: relating fundamental studies on structure, morphology and reactivity to industrially relevant processing and product development strategies. By leveraging in-depth analytical methods, exercising precise control over supramolecular interactions, understanding structure-reactivity aspects, and maintaining a holistic focus on techno-economics and circularity, we can harness the promise of this important renewable feedstock, establishing lignin’s key role in future bio- and circular-economy strategies.

## Abstract 2

These concluding remarks summarise the *Faraday Discussion* that was held in London, United Kingdom, on “Frontiers in physical chemistry for lignin valorisation”, between the 10th and 12th of September 2025.

## Graphical abstract

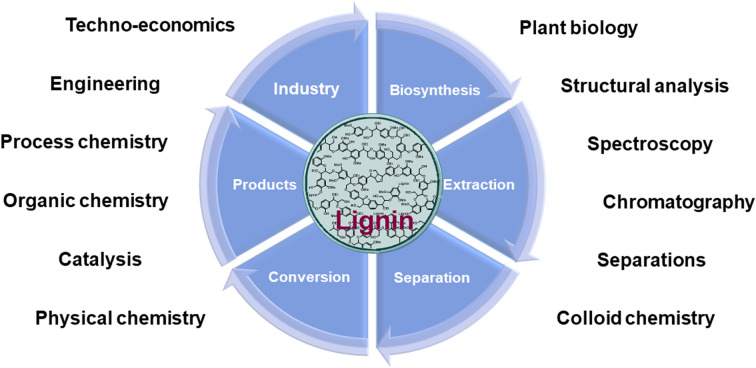

## 1. Introduction: A brief historical perspective

It is a distinct pleasure and honor to summarize and frame the proceedings of the recent *Faraday Discussion* on Frontiers in physical chemistry for lignin valorisation. Having arrived at this meeting with ample excitement and anticipation, it was very rewarding to witness all the recent progress made by our community, both in terms of fundamental understanding as well as industrial relevance.

Due to its unique, somewhat mysterious and recalcitrant structure, the aromatic biopolymer lignin has fascinated chemists for decades.^1^ Starting from the beginning of the 20th century, research was mainly focused on understanding the composition of wood, and elucidating the chemical structure of lignin in this context. Notably, much of this work was carried out without readily available spectroscopy methods. Hence, most of these classical studies focused on the isolation of lignin from the lignocellulose matrix, scrutinizing its reactivity and assessing the chemical nature of the resulting fragments.^2–4^ Later, 2D NMR and other specialized spectroscopic and chromatographic methods, in conjunction with plant biology studies and biosynthetic considerations, helped to uncover new structural insights, including differences between lignins of various plant origins.^5–7^

Historically, paper and pulp production has been the major driving force behind industrial lignocellulose valorization, producing significant amounts of highly condensed lignin side-products, which were first discarded and later fully integrated into the process as a low-value energy source.^8^ In addition, the oxidative conversion of kraft lignin was implemented and scaled up, albeit with low vanillin yield.^9^

It was with the advent of bioeconomy strategies (early 2000s) that the scientific community regained an unparalleled interest in lignin. Now, the focus has shifted towards the efficient valorization of lignin as a source of aromatic chemicals and materials in the context of establishing innovative biorefinery concepts.^10–13^ As a result, efficient catalytic depolymerization to aromatic building blocks has emerged as a central goal. Despite concentrated efforts from the broader catalysis field, this has turned out to be very challenging, due to the recalcitrant nature of lignin and undesired recondensation phenomena during fractionation and depolymerization.

‘Lignin-first’ strategies have emerged as a key enabling solution to the depolymerization problem.^14,15^ These methods apply catalytic or protection group stabilization in order to minimize undesired recondensation phenomena, and deliver either aromatic monomers in markedly improved yields and high selectivity, or protected lignins, depending on the type of stabilization.^16–18^ At the same time, these methods maintain the value of the cellulose and hemicellulose.

The ‘lignin challenge’ has captivated various scientific communities to deliver new solutions for lignin valorization. Much progress has been made in chemical and biochemical engineering, advancing fractionation in batch and flow,^19^ and in alternative solvents;^20^ depolymerization has been addressed (bio)catalytically, electrochemically and photochemically;^21^ new polymers and materials,^22–24^ including LNP-based nanomaterials, have been designed from lignin and derived depolymerization products; new structural insight has been gained through advanced imaging and spectroscopic methods; and progress has been made in the plant biology and biotechnology domains.

To conclude, lignin, the only high-volume, naturally occurring aromatic polymer, represents an unparalleled opportunity for sustainable material and chemical production. Yet, it remains the “dark horse of biorefining” – a source of immense promise, but one that has yet to yield a major, large-scale product on the market.

## 2. This *Faraday Discussion*

The discussions held across the four sessions were deliberately structured to confront the key challenges that have historically stalled valorisation, including strides towards deeper structural understanding, rapid elucidation of complex product mixtures, mitigating lengthy analysis protocols, studying catalyst deactivation, and identifying strategies to access suitable lignin-based products.

The work presented during this meeting illustrated how overcoming these hurdles demands a multidisciplinary approach. We need to sharpen our research questions and increase our fundamental understanding across a diverse circle of disciplines to successfully move the field forward and finally tame the “wild horse” of biorefining.

### 2.1. Spiers Memorial Lecture

After a welcome by **Professor Roberto Rinaldi**, the meeting kicked off with the Spiers Memorial Lecture by **Dimitris Argyropoulos**. His lecture, titled “Spiers Memorial Lecture: organic, physical & polymer aspects pivotal in lignin valorization”, addressed four key thematic areas (https://doi.org/10.1039/D5FD00108K). The first area examined important chemical transformations in lignin, that occur during fractionation and lignocellulose processing, and the emerging structural understanding as a consequence of the processing conditions, with a special focus on kraft lignin. The second part addressed fractionation strategies, stressing their importance in reducing the polymer’s inherent heterogeneity towards more uniform lignin fractions, thereby sharpening molecular weight distributions, increasing structural understanding, and ultimately enabling better property and performance control of derived products. Overall, this approach delivers more suitable starting materials to enable practical applications. Next, the discussion emphasized the importance of fundamentals in the chemical and physical behavior of lignin in solution, specifically its self-assembly processes driven by supramolecular interactions, such as π–π stacking and hydrogen bonding, which are crucial for designing lignin-based nanomaterials for applications in sectors like agriculture and packaging. Finally, the lecture projected a broader future vision for lignin valorization, especially focusing on its rheological and antioxidant properties in polymer blends and its potential as a non-petroleum precursor for carbon fiber production, while critically assessing barriers such as low molecular weight and thermal behavior that hinder effective fiber formation.

On a personal note, I would like to extend my warmest congratulations to Prof. Argyropoulos on his Spiers Memorial Lecture. During my own post-doctoral studies, and as a newcomer to this field, I immediately came across his landmark papers on lignin structure and analysis, which were truly valuable and educational, shaping my own understanding of this field.

### 2.2. Session 1: Native lignin solvation and extraction

The five talks in Session 1 explored the physical chemistry of lignin, focusing on the fundamental forces driving its self-assembly, the stability challenges during its extraction, and the role of computational chemistry in elucidating both fractionation and self-assembly processes. The session began with a focus on the fundamental structure and preparation of lignin-based nanomaterials.

**Malin Wohlert** presented an analysis of π–π stacking interactions in lignin nanoparticles using molecular dynamics (MD) simulations. This research demonstrated that a specific peak in the wide-angle X-ray scattering (WAXS) profile, often attributed to sandwich π–π stacking, was actually caused primarily by other intramolecular structural motifs involving the α-carbon and ring carbons. This is interesting and significant for a better understanding of aggregation phenomena, as they are strongly driven by weak interactions. The development of new methods to enable rapid insight into such interactions is truly valuable. This contribution has shown that combining X-ray scattering with molecular modelling can provide important novel insights into the interpretation of WAXS profiles, thereby addressing the need for facile analysis, and ultimately leading to a better understanding of aggregation phenomena.

Lignin nanoparticle (LNP) formation was among the most discussed topics during this meeting. The community wishes to gain more fundamental knowledge of formation, aggregation and assembly of LNPs and understand the effect of processing conditions and isolation procedures on LNP formation. To address the latter, **Juan Carlos Dominguez** systematically compared different preparation methods for LNPs, including different fractionation methods and both bottom-up and top-down approaches. The work showed that while ionosolv fractionation provided much higher LNP yields (precipitation yield of 56.6–63.1% compared to 9.0–19.2% for organosolv), organosolv fractionation yielded LNPs with better sphericity and less aggregation. The most spherical LNPs were achieved by applying a bottom-up precipitation strategy (progressive, controlled antisolvent addition) to the organosolv liquors. The markedly different LNP morphologies obtained during these experiments are striking. On one hand, this opens the path towards more precise morphology control by careful selection of suitable process and precipitation conditions; on the other hand, concerns could be raised about batch-to-batch variations in LNP yield and morphology, and reproducibility in general, when comparing work across different laboratories.

After a break, the session then transitioned to excellent computational work addressing scalable extraction and valorization, especially regarding the nature and stability of different lignin–carbohydrate complexes, and related implications in lignocellulose processing. The kraft pulping process removes a large proportion of the lignin present in wood, but not all of it. Hence, subsequent bleaching steps are required to degrade the residual aromatic residues to achieve acceptable levels of brightness. These residual lignin fragments must originate from lignin–carbohydrate complexes which feature more stable covalent linkages than the typical β-O-4 moiety. To gain more insight, **Thomas Elder** addressed the systematic assessment of bond dissociation energies (BDEs) of various relevant lignin–carbohydrate complexes (LCCs) using density functional theory (DFT) calculations. It was found that α-benzyl ethers and γ-esters are the most stable LCCs, possessing the highest homolytic BDEs (≈80 kcal mol^−1^), while phenyl glycosides were markedly less stable (≈60 kcal mol^−1^). This difference in stability supports the hypothesis that homolytic cleavage is the dominant degradation mechanism under biorefinery conditions and agrees well with reported experimental data.

**Scheme 1:**
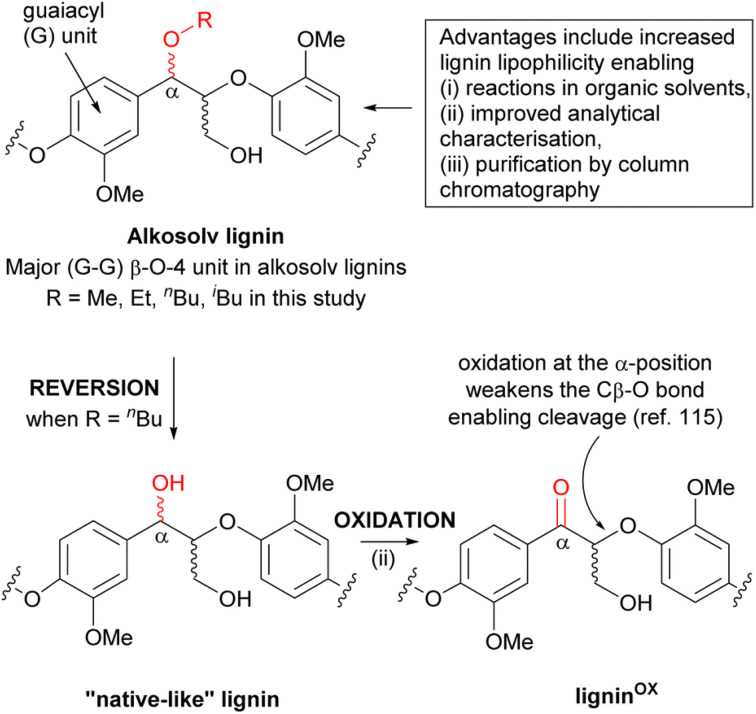
The presence of the α-modified β-O-4 linkage in alkosolv lignins provides several advantages but a key disadvantage arises from the inability to oxidise the α-position to the corresponding ketone. This oxidation is known to facilitate depolymerisation of the lignin. One solution is to convert the alkosolv lignin back to a “native-like” lignin by reinstalling the α-OH into the β-O-4 linkage (a process referred to as reversion). Reproduced from Davidson *et al.*, *Faraday Discuss.*, 2026, DOI: 10.1039/D5FD00074B with permission from the Royal Society of Chemistry.

Next, **Nicholas Westwood** presented exciting work directed towards scalability of organosolv processing involving a range of aliphatic alcohols as fractionation solvent, and exploiting the locally important Sitka spruce sawdust. The study systematically compared pretreatments, and identified butanosolv and pressurized methanosolv as the most economically viable choices, based on the lignin value factor (LVF) introduced therein. Notably, this method allowed for both a very high alkosolv lignin yield and an excellent retention of native β-O-4 linkages *via* alcohol incorporation into the β-O-4 moiety, resulting in (partially) ether-protected organosolv lignins (Scheme 1). Moreover, the obtained product could be reverted back to the ‘native-like’ lignin structure, followed by selective oxidation in the α-position, leading to lignin^OX^. This process was successfully scaled up to produce lignin^OX^ on a >50 g scale, demonstrating a viable path for valorization. The remarkable isolated yield and β-O-4 retention should raise discussion about the nature of this process. In principle, α-protection *via* ether formation could be regarded as a distinct type of ‘lignin-first’ approach. The efficiency of the butanosolv process is probably not only connected to reactivity but also to the distinct physical–chemical solvent parameters that butanol provides to solubilize and remove butanol-protected lignin from the lignocellulose matrix.

**Fig. 1:**
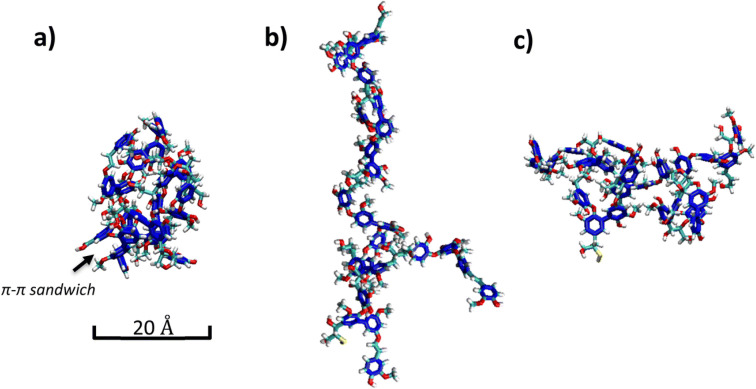
Representative snapshots from classical molecular dynamics simulations. AIKL molecule (a) in a vacuum, (b) in DMSO and (c) in ethylene glycol (colours: dark blue – aromatic rings of AIKL; for atoms: cyan – C, red – O, white – H, yellow – S). Reproduced from Sgarzi *et al.*, *Faraday Discuss.*, 2026, DOI: 10.1039/D5FD00076A with permission from the Royal Society of Chemistry.

The session concluded by returning to the core physical chemistry of LNP formation. **Massimo Sgarzi** presented an interesting and detailed look at the supramolecular interactions in softwood kraft lignin nanoparticles prepared by the solvent–antisolvent method. Molecular dynamics simulations confirmed that intramolecular and intermolecular H-bonds, together with π–π stacking, are the major driving forces for nucleation. They demonstrated that LNP size is a kinetic function, with the slow addition of solvent or low initial concentration minimizing aggregation numbers and hydrodynamic volume of the resulting LNPs (Fig. 1). Interestingly, it was also found that the colloidal stability of the LNPs was primarily governed by the superficial concentration of carboxyl and condensed guaiacyl units. The contribution sparked detailed discussion about LNP phenomena. LNP formation is strongly dependent on the interaction of the lignin with the solvent. Also, since secondary interactions are very important in initial nucleation and aggregation phenomena, these may be dependent on the lignin structure, which in turn depends on the biomass source, as well as processing conditions. It is clear that this work opens many interesting avenues for future studies with the aim of elucidating important structure–property relationships and achieving more precise control over LNP morphology.

### 2.3. Session 2: Lignin stabilisation and degradation in lignocellulosic fractionation processes

The second session provided a comprehensive overview of controlling lignin’s reactivity and structure, ranging from fundamental electrochemical tracking to industrial-scale catalytic optimization. The talks highlighted the crucial link between process engineering and product quality.

**Fig. 2:**
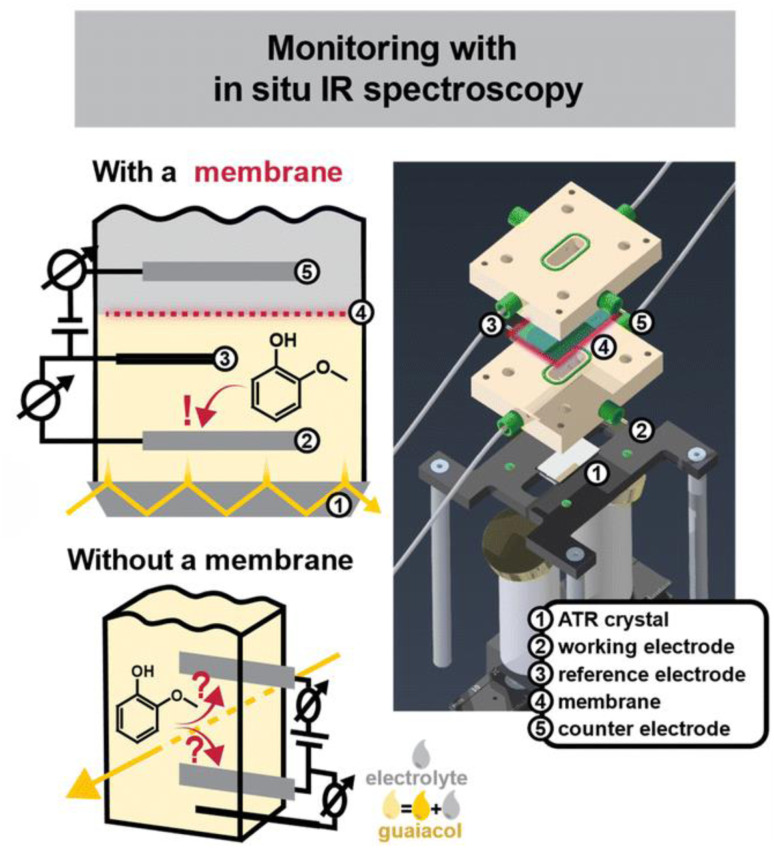
Schematic diagram of a two-chamber *in situ* spectro-electrochemical cell in attenuated total reflection (ATR) configuration, alongside a 3D rendering used for its construction. Unlike conventional commercial cells in transmission configuration, the design incorporates a membrane that separates the working and counter electrodes, preventing unintended reactions at the counter electrode and thus potentially misleading mechanistic insights. Reproduced from Schwartmann *et al.*, *Faraday Discuss.*, 2026, DOI: 10.1039/D5FD00069F with permission from the Royal Society of Chemistry.

**Sibylle M. K. Schwartmann** opened the session by addressing the elusive intermediates formed during lignin electro-oxidation. The intriguing setup presented featured a novel, two-compartment spectro-electrochemical cell that integrates *in situ* attenuated total reflectance-infrared (ATR-IR) spectroscopy (Fig. 2). Crucially, the cell uses a membrane to separate the working and counter electrodes, preventing product crossover and unintended side reactions that can lead to misleading mechanistic data. Using guaiacol (β-O-4 model monomer), the *in situ* ATR-IR spectroscopy provided real-time tracking, confirming the formation of oxidized species including quinones, catechols, dimers and oligomers (Fig. 2). Undoubtedly, there is a need for such *in situ* monitoring of electrochemical processes, a rarity in the lignin field. This should significantly increase our mechanistic understanding and ultimately shed light on new intermediates and recondensation phenomena during lignin depolymerization. This also necessitates the use of more realistic lignin-models. Attempting this analysis with real organosolv lignin streams would be a fantastic future exercise, both highly challenging and with distinct novelty and utility.

**Heiko Lange** presented an elegant engineering solution for the persistent challenge of purifying heterogeneous technical lignins. The approach utilized a specialized pump-assisted Soxhlet extraction system, which allowed for the continuous use of non-azeotropic solvent mixtures (*e.g.*, aqueous acetone). This capability enabled fine-tuned control over the solvent’s polarity and solubility parameters, resulting in superior fractionation compared to conventional methods. The team successfully separated softwood kraft lignin (SKL) into fractions that were structurally uniform but differed only in molecular weight, and cleanly isolated a high-purity lignin–carbohydrate complex (LCC) fraction from organosolv lignin. This fascinating smart and simple technique offers a scalable and low-cost pathway to generate homogeneous, high-value lignin streams.

**Fig. 3:**
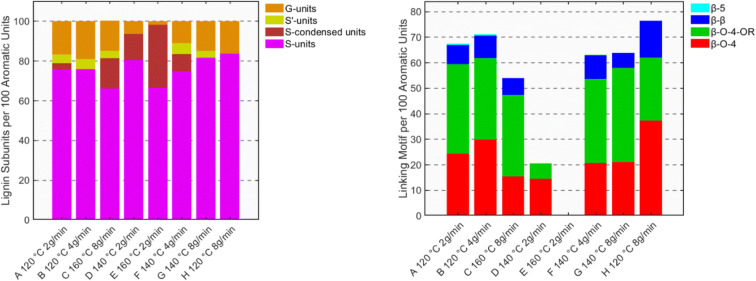
Distribution of lignin linking motifs (left) and lignin subunits (right) quantified from 2D-HSQC NMR semi-quantification for the runs (A to H). Reproduced from Appiah *et al.*, *Faraday Discuss.*, 2026, DOI: 10.1039/D5FD00104H with permission from the Royal Society of Chemistry.

In the realm of efficient lignocellulose processing, we heard an interesting contribution from **Amponsah Preko Appiah** from the **Deuss** group, highlighting the advantages of an integrated modeling–experimental approach in lignocellulose fractionation processes. Using a semi-continuous flow-through ethanosolv reactor and response surface methodology (RSM), they precisely determined the ideal conditions for birch wood extraction. The results pointed to the synergistic interplay between high temperature (for maximum yield) and a high solvent flow rate (for structural integrity). The rapid flow rate acts as a chemical stabilizer, quickly flushing reactive lignin fragments from the reactor and thereby preserving the vulnerable β-O-4 linkages and preventing repolymerization (Fig. 3). The optimized process achieved 82 wt% delignification with high structural retention, cutting the time to 30 minutes and reducing solvent consumption by 40%. Conceptually connecting this study with the butanosolv extraction work presented by **Westwood**, structural stabilization is not only provided by the ‘flow effect’ but also by the incorporation of ethanol into the native β-O-4 moieties.^25^ The high yield and structural integrity of the obtained lignin point towards a ‘lignin-first’ approach using alcohols.

**Fig. 4:**
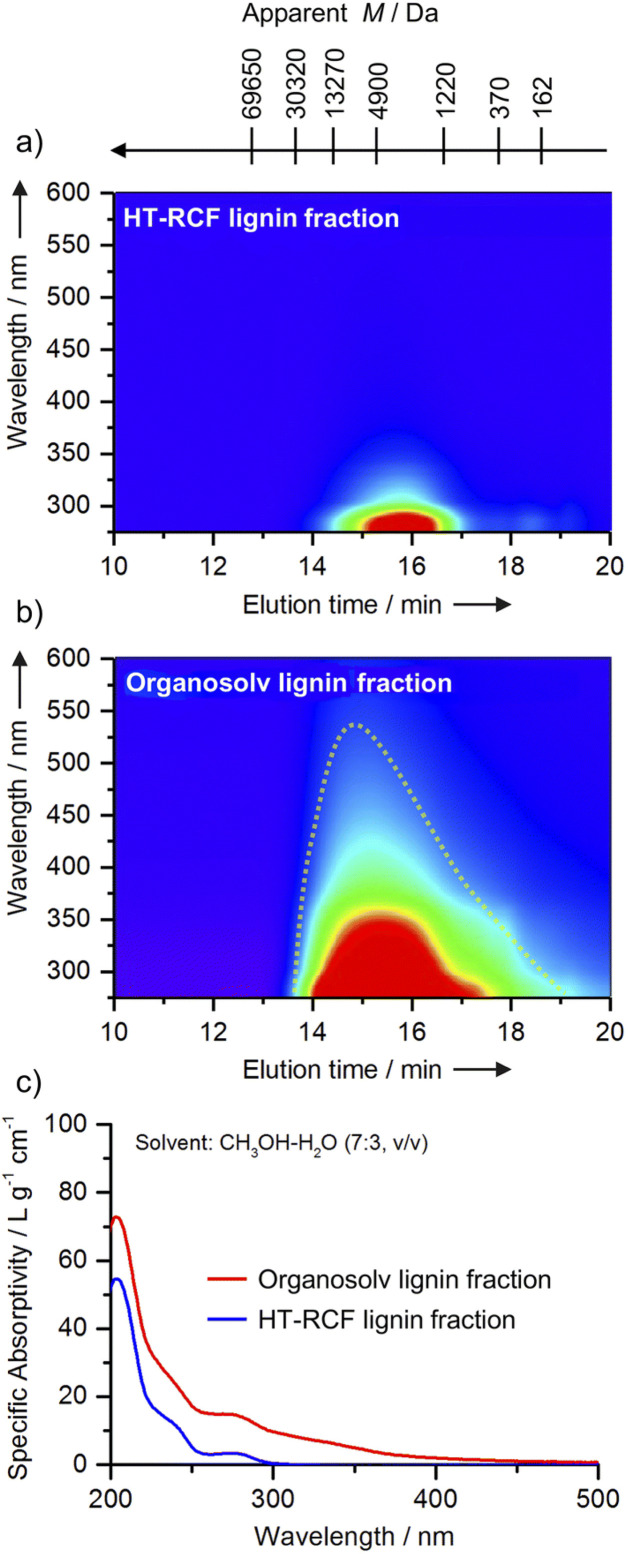
UV-Vis-resolved GPC analyses provide a comparative assessment of the HM fractions derived from HT-RCF lignin and organosolv lignin. All GPC measurements were performed using 0.1 wt% LiBr/DMF as the eluent. In panel (a), the HT-RCF fraction exhibits pronounced absorption within the ultraviolet region (280–300 nm), while absorbance above 320 nm remains minimal. In contrast, panel (b) reveals that the organosolv fraction displays a broader absorption spectrum, extending from 280 nm well into the visible region (up to 550 nm). Panel (c) presents overlaid UV-Vis spectra for these fractions, measured in CH_3_OH–H_2_O (7 : 3, v/v). This comparison clearly illustrates a strong long-wavelength absorption tail for organosolv lignin (red curve) compared to the HT-RCF lignin (blue curve). Such spectral differences are indicative of an increased population of conjugated chromophores within the organosolv sample. These findings highlight the substantial impact of HT-RCF processing on the decrease in chromophore content and the distinguished electronic structure of the resulting lignin fractions. Reproduced from Gao *et al.*, *Faraday Discuss.*, 2026, DOI: 10.1039/D5FD00109A with permission from the Royal Society of Chemistry.

Along the lines of lignin-first processing and toward optimizing the reductive catalytic fractionation (RCF) processes, **Roberto Rinaldi** introduced a powerful combined analytical tool for monitoring such systems, with a special focus on catalyst deactivation phenomena. The talk detailed the development of the spectral index (SI_320_), a rapid, concentration-independent metric derived from gel permeation chromatography (GPC)-UV-Vis mapping (Fig. 4). The SI_320_ tracks the formation of chromophores (molecules that cause darkening and condensation, absorbing at ≥320 nm) relative to the aromatic signal (280 nm). By generating SI_320_ (M) profiles across the lignin’s molecular weight, the method provided an early warning system for catalyst deactivation. It detected the subtle loss of stabilization activity in the heavy oligomer fraction well before conventional bulk yields showed any decline, allowing for proactive process management. Given that catalyst stability is central to the viability of lignin-first biorefineries, and that conventional characterization often fails to detect the subtle deactivation processes that govern product quality, the elegant combination of UV-Vis spectroscopy with gel permeation chromatography (GPC) resulted in a powerful method for rapid analysis of RCF phenomena and a sensitive diagnostic of hydrogenation performance decline in reductive catalytic fractionation (RCF) processes. Interestingly, determining molecular weight in parallel with UV-Vis detection also provided new insight into the chemical nature of hydrogenated, yet still unconverted, lignin oligomer species, which were not discussed previously.

The last contribution to the session, by **Ling-Ping Xiao**, focused on reductive catalytic fractionation of lignocellulose and the importance of catalyst design to increase the feasibility and scalability of such processes. While Cu-based heterogeneous catalysts have been previously established for RCF,^26^ hydrogenolysis or catalytic hydrothermal processes,^27^ the rational design of base-metal catalysts to improve catalyst stability and product selectivity is still urgently needed. This interesting work engaged in the rational design of an efficient, non-precious-metal catalyst based on CuO nanoparticles supported on monoclinic ZrO_2_ (CuO/m-ZrO_2_), and successfully leveraged the synergistic effect of the metal sites and the ZrO_2_’s acid sites to improve hydrogenolysis efficiency. Under optimal conditions, the catalyst delivered an impressive 18.7 wt% yield of monophenols from softwood lignin, with high selectivity (82.3%) for 4-*n*-propanol guaiacol (Pol-G). Given this impressive yield, the question could be raised whether the catalyst is indeed able to cleave bonds other than C–O linkages. The research confirmed the reaction pathway and demonstrated the practical potential of the catalyst through a successful gram-scale reaction, achieving 68.3% of the theoretical monomer yield.

### 2.4. Session 3: Commercially available technical lignins

Session 3 provided a series of practical and fundamental breakthroughs, illustrating strategies to convert industrial lignin streams into high-value products by carefully controlling properties from the molecular level in solution to the final composite or coating. The session successfully addressed key commercial hurdles, particularly odor, solubility, and color.

**Fig. 5:**
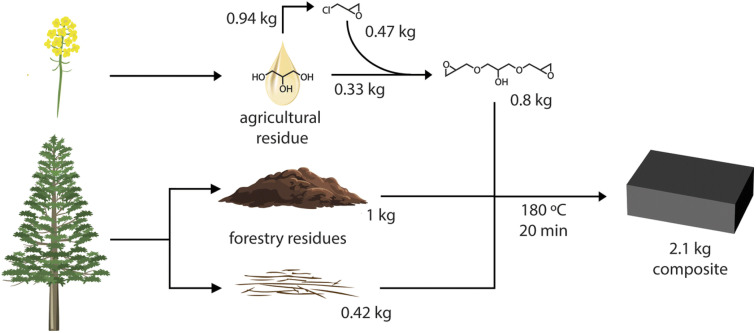
Residues from agriculture, glycerol from fatty acid methyl ester production, are used to produce glycerol diglycidyl ether, which is combined with the precipitated kraft lignin and wood fibers that generate a composite material that can substitute bisphenol A. Reproduced from Orebom *et al.*, *Faraday Discuss.*, 2026, DOI: 10.1039/D5FD00053J with permission from the Royal Society of Chemistry.

The session commenced with **Alexander Orebom** who presented a solution to the long-standing problem of odor and performance in lignin-based composites with their new industrially relevant resin, LigniSet®. The researchers successfully developed an odor-free, 100% bio-based thermoset by combining unmodified kraft lignin (50% content) with the sustainable component glycerol 1,3-diglycidyl ether (GDE) (Fig. 5). This direct synthesis method avoids the costly and complex pre-fractionation or multi-step chemistries typically required for high-performance materials. The resulting resin exhibited remarkable mechanical properties (*e.g.*, flexural strength up to 47 MPa when reinforced with wood fiber) and was shown to be recyclable without any loss in performance, addressing a critical sustainability metric. Furthermore, a life cycle assessment (LCA) revealed that using LigniSet® as a substitute for conventional petroleum-based resins, such as bisphenol A diglycidyl ether (DGEBA), resulted in a significant net-negative climate change impact, confirming that valorization offers substantial environmental benefits over simply burning the lignin for energy. Given the long-standing challenge and problem of odor in kraft-lignin-based applications, it is needless to say that this contribution sparked a lot of discussion related to the origin of the odorless product and the molecular level understanding of these phenomena.

**Fig. 6:**
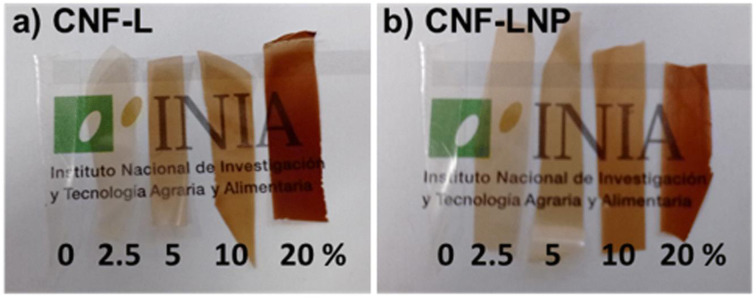
Nanocellulose–lignin films with bulk lignin (a) and lignin nanoparticles (b). The lignin content of each film is indicated in the bottom of the images. Reproduced from Martín-Sampedro *et al.*, *Faraday Discuss.*, 2026, DOI: 10.1039/D5FD00063G with permission from the Royal Society of Chemistry.

Following this, **Raquel Martín-Sampedro** and **Antonio Ovejero-Pérez** presented work on “The use of kraft lignin to enhance nanocellulose film properties”, focusing on creating high-performance active food packaging. Their research compared two methods of incorporating kraft lignin into nanocellulose (CNF) films: adding bulk lignin *versus* forming lignin nanoparticles (LNP) *in situ*. They demonstrated that the LNP protocol was decisively superior. While bulk lignin caused aggregation and degraded the mechanical and barrier properties at high concentrations, LNPs conferred significantly greater UV-shielding and antioxidant capacity, while simultaneously leading to enhanced water vapor barrier properties and higher bulk density in the final film (Fig. 6). These findings established that *in situ* LNP formation is a highly effective and scalable method for producing bio-nanocomposite films with properties rivaling traditional plastics.

The focus then shifted to the fundamental chemistry of processing with a talk covering the physicochemical properties of lignin solutions in aqueous sodium hydroxide by **Veronica Calado**. The researchers sought to bring quantitative control to the critical industrial step of dissolving lignin in aqueous sodium hydroxide (NaOH). Using a central composite design (CCD), it was investigated how four key factors (time, temperature, NaOH concentration, and the lignin-to-alkali ratio) impacted solution density, mass fraction, and pH. Their robust empirical models confirmed the joint action of high alkali concentration and elevated temperature in driving molecular aggregation and conformational changes in lignin. Crucially, their work demystified a widely accepted statement by showing that the mass fraction of solubilized lignin displayed a maximum value, and could actually decline with excessive NaOH addition when a fixed lignin mass was used. This demonstrated that solubility depends on more complex factors than simply the OH^−^ to phenolic-OH ratio or pH.

**Fig. 7:**
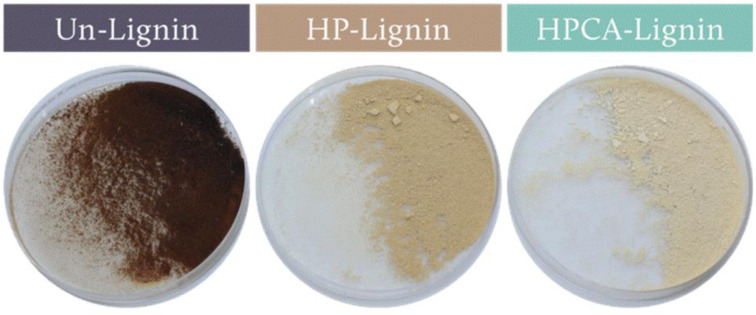
Visual appearance of bulk unmodified lignin (Un-Lignin), lignin oxidized by the hydrogen peroxide method (HP-Lignin), and lignin oxidized by the peroxy-citric acid method (HPCA-Lignin). Reproduced from Meneses *et al.*, *Faraday Discuss.*, 2026, DOI: 10.1039/D5FD00072F with permission from the Royal Society of Chemistry.

The session concluded with **Camila A. Rezende** who addressed another major commercial limitation: lignin’s intense, dark color. The team employed oxidation methods to create light-colored lignin for use in color-sensitive applications, specifically protective coatings for artifacts such as white paper. They compared hydrogen peroxide (HP) oxidation with a novel peroxy-citric acid (HPCA) treatment, finding that the HPCA method achieved the most profound whitening and highest chromatic neutrality (Fig. 7). When formulated as nanoparticles (LNP), this lightened lignin imparted strong UV-shielding and antioxidant capacity to nanocellulose coatings with minimal visual interference, showing that lignin’s functional benefits can be harnessed for high-end applications like cultural heritage conservation without compromising aesthetic integrity.

### 2.5. Session 4: Strategies for compatibilisation of extracted lignin properties with commercial applications

Session 4 focused on bridging the gap between extracted lignin and high-performance commercial applications, detailing sophisticated strategies for chemical modification, precise tuning of molecular properties, and advanced material processing to meet industrial standards in areas such as epoxy resins, anticorrosive coatings, and thermoplastic materials.

The session opened with an engaging talk by **Mojgan Nejad** on the in-depth analysis of kraft lignin epoxy thermosets, which directly addressed the goal of replacing the toxic petroleum-based compound bisphenol A (BPA) in epoxy resins. The research demonstrated the successful synthesis of epoxidized lignins using a sustainable platform. The resulting lignin-based resins exhibited significantly faster curing kinetics (lower activation energy) and the hardwood lignin system (E-HW) showed the best overall thermomechanical properties due to its high functionality. This approach, as well as the analysis effort towards better understanding of kraft lignin epoxy thermosets, is remarkable and very valuable for future industrial implementation of such bio-based thermoset materials. The manufacturing of epoxy thermosets is perhaps one of the most promising industrially relevant directions.

Following this, the focus shifted from bulk epoxy resins to coatings with **Narayanan Rajagopalan’s** presentation on molecular-weight-dependent water uptake and dynamics in lignin-based epoxy anticorrosive coatings. The study systematically compared high- and low-molecular-weight (*M*_w_) lignin fractions as pigments. The key finding was the superior barrier performance of the low-*M*_w_ solvent-fractionated lignin (KL-EtOAc-EP), which exhibited the lowest equilibrium water uptake and lowest diffusion coefficients. This was attributed to the lower-*M*_w_ lignin minimizing supramolecular aggregation, leading to enhanced dispersion and better integration within the epoxy matrix.

After a refreshment break, the session resumed with **Agnieszka Brandt-Talbot** who discussed advanced processing techniques. Her talk highlighted how hot-drawing ionic-liquid-spun lignin–poly(vinyl alcohol) fibres increases strength and polymer alignment. Focusing on creating precursors for low-cost sustainable carbon fibres, the work demonstrated that applying hot-drawing to lignin–poly(vinyl alcohol) (PVA) composite fibres achieved exceptionally high draw ratios (up to 20). This process significantly increased the fibre tensile strength (up to 249 MPa) by inducing oriented crystalline PVA domains. However, this beneficial molecular alignment was lost during the subsequent slow oxidative stabilisation step. The ample discussion following this contribution highlighted the potential of this method towards industrial implementation.

**Fig. 8:**
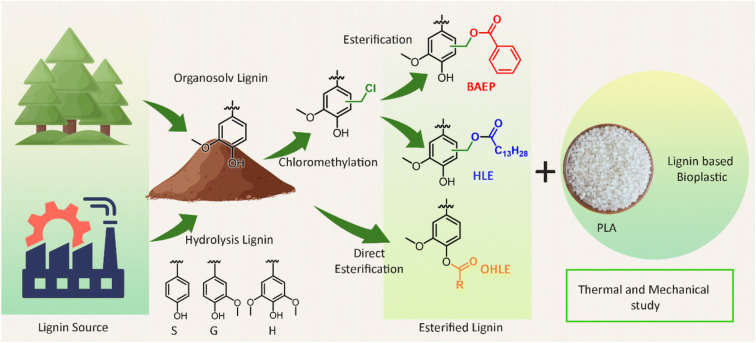
Esterification of organosolv pine lignin and hydrolysis lignin in three different schemes and their compatibility study with PLA. Reproduced from Mohan *et al.*, *Faraday Discuss.*, 2026, DOI: 10.1039/D5FD00068H with permission from the Royal Society of Chemistry.

The final presentation of the session addressed thermoplastic applications, with **Yevgen Karpichev** detailing how esterification significantly affected the polymer matrix properties of lignin-based composites. The strategy involved systematic chemical modification of lignin through esterification to control its compatibility with polylactic acid (PLA) (Fig. 8). The research showed that hydrolysis lignin ester (HLE) and benzoic acid ester (BAEP) were effective reinforcing agents, increasing PLA’s stiffness and thermal stability. Conversely, the long-chain C16 ester acted as a strong plasticizer, significantly increasing the PLA composite’s ductility and flexibility, while lowering its *T*_g_ (to 55 °C), demonstrating the potential for precisely tailoring bio-based material properties. This work not only underscores the industrial potential of this approach but also represents a remarkable advancement, where precise structural understanding and manipulation of the lignin structure is possible in a controlled manner, which is highly relevant.

## 3. Conclusions

### 3.1. Lignin: a creative, fundamental scientific exercise

Lignin offers amazing avenues for creative fundamental scientific research. The knowledge that has already been created opens new possibilities for deeper investigations in diverse areas. For example, we have yet to fully understand the structure of lignin and its structural changes, develop rapid, reliable and comprehensive analytical tools, and recognize the nature, role and reactivity of functional groups as well as free radicals as a consequence of variable structure. The various functional groups and derived fragments might inspire the development of novel sustainable (bio)catalytic, photo- and electrochemical transformations and new catalysts, addressing the conversion of C–C, C–O and C–H bonds. Elaborate engineering solutions can be developed for fractionation, processing and depolymerization, and isolation. Physical chemistry studies are needed to uncover the nature of secondary interactions, aggregation and self-organization phenomena, and the formation of colloidal systems.

To revolutionize the valorization of lignin with regards to integration into the circular economy, we need to achieve control over structure, reactivity, selectivity, lignin-based material properties and recycling.

Lastly, it is important to emphasize that lignin is ideally positioned to create new knowledge at **disciplinary interfaces**, involving, for example, biotechnology; batteries and energy storage systems; hybrid structures and nanomaterials; circular polymers; materials and composites; as well as engineering and advanced analysis across length scales (from the nanoscale to the macroscale), and to integrate computational chemistry, machine learning and artificial intelligence domains.

### 3.2. Establishing best practices, and standardization of protocols

Lignin’s intriguing and rather complex structure offers various new research opportunities, yet raises major challenges with regards to standardization of research protocols across multiple domains.

**Fig. 9:**
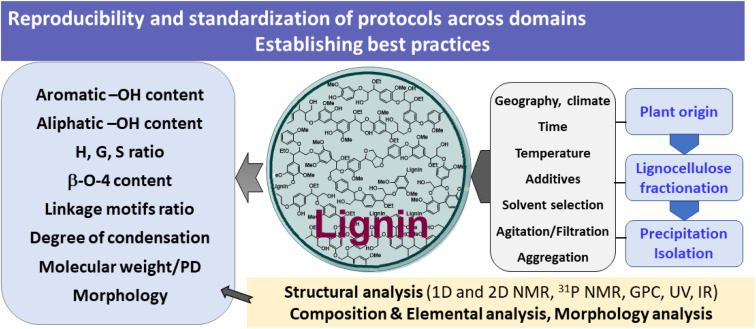
Processing and structural considerations of lignin: the need to establish best practices and standardized protocols across domains.

The structure of lignin depends on the wood species, its geographical origin, and even environmental conditions. Most laboratories will perform some sort of fractionation and lignin isolation protocol, which is followed by structural characterization. It is known that the fractionation conditions (temperature, pressure, solvent, additives, and time) may strongly influence the structure of the obtained lignin. Similarly, the isolated yield is strongly affected by the precipitation protocols (time, solvent mixtures, temperature, filtration, and agitation). Moreover, aggregation phenomena and the formation of soluble colloidal lignins, which depend on the solvent systems, strongly influence the extent of lignin precipitation (Fig. 9).

In summary, given the large number of academic papers, there is a need to establish best practices in lignin fractionation, isolation and structural characterization protocols, as well as the conversion of lignin downstream and analysis of the derived product mixtures. Moreover, in analogy to the characterization of organic compounds, we need to identify basis parameters and key analysis techniques, specifically working out guidelines for minimum acceptable parameters for the structural analysis of lignin. We must be able to ensure batch-to-batch reproducibility of data, as well as comparability of the obtained results between different laboratories.

### 3.3. Hitting the market

Perhaps all participants in this meeting will agree that the old phrase ‘*you can make anything out of lignin except money*’ can be laid to rest in light of recent scientific and technological innovations in this field. In fact, the new ‘(multi)million-dollar question’ is: *Can we bring lignin-based chemicals or materials to the market at substantial scale?* And which products are the first to be targeted? Achieving such a breakthrough would consolidate lignin’s central position in global bioeconomy efforts, and boost the profitability of refineries, some of which currently produce lignins at an industrial scale.

Our scientific community is clustered around various domains (*e.g.* kraft lignin *vs.* lignin first, chemicals *vs.* polymers/materials) and it is gratifying to witness so many diverse creative efforts. In my opinion, it is necessary to pursue multiple distinctly different directions to expand our future horizons; and it is perfectly reasonable to differentiate between short-term and long-term strategies, especially in terms of industrial implementation.

**Fig. 10:**
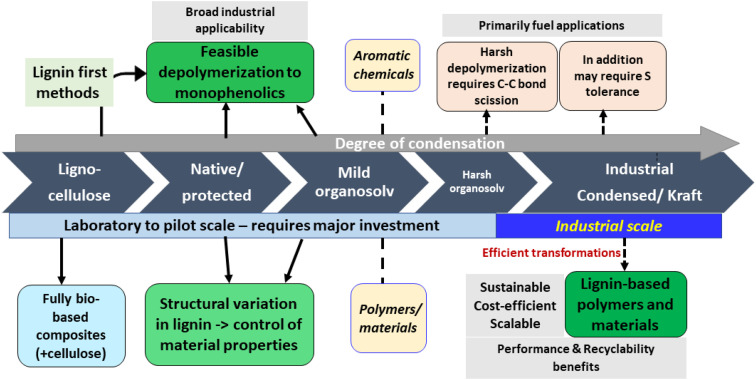
Potential application areas of lignins depending on degree of condensation and industrial availability.

Based on the discussions during the meeting, the efficient valorization of lignins, which are produced on an industrial scale and currently not exploited to their full potential, appears to be the most urgent task to address (Fig. 10). At the moment, this primarily includes kraft lignins.^28^ Given the relatively condensed nature of such lignins, finding breakthrough applications in the materials and polymers sector seems to be the most sensible target. Ideally, such materials would display performance advantages and recyclability benefits compared to existing petrol-based products, and should be produced in a feasible, scalable, sustainable and cost-effective manner. It is important also to consider the balance between feedstock availability and intended production volumes. Constant and open dialogue between industry and academia has to identify desired performance indicators, scalability challenges, and suitable application areas for implementation. For these highly condensed lignins, depolymerization appears to be less feasible, at least on the short term. Fundamental research in catalysis and engineering first needs to convincingly address the cleavage of strong C–C linkages and overcome sulfur intolerance; the resulting mixtures could then be funneled, for example, into high-volume fuel/naphtha applications. Electrochemical conversion of condensed lignins to aromatics represents another interesting direction, pending scalability aspects.

Another, conceptually different direction is to fundamentally rethink biomass fractionation, and to establish and upscale biorefineries that operate based on entirely novel concepts. Such conceptual changes in the fractionation method (*e.g.* mild organosolv) have a significant impact on the structure of the resulting lignins, so that they are more amenable to depolymerization or afford aromatics directly *via* specific lignin-first processing methods. Despite a longer-term effort, these approaches harbour the most potential to enable sustainable access to aromatic chemicals from lignin. Such new biorefineries should be implemented from the bottom up, in parallel with classical, industrial pulping methods, since established industries are unlikely to switch their existing technologies. Therefore, this would require considerable early investment, including government subsidies, and/or involve spin-off activities. In fact, successfully exploiting these avenues, several new technologies have already reached the pilot stage (Bloom, Biocon, Vertoro). Given the obvious demand for renewable aromatic chemicals in several industrial sectors, this approach would unlock reliable access to bio-aromatics, and open an entirely new development space toward the production of valuable chemicals and materials. Well-defined aromatic platform chemicals would provide precise control over synthetic strategies and product properties, with the possibility of embracing green chemistry and circularity concepts by design.

Broadly speaking, the technology landscape is diverse and rapidly evolving, and it will be exciting to witness also many unexpected new innovations involving lignin. The participation of industrial partners and emerging investment strategies will be crucial, especially in line with bio-based economy and circular economy strategies.

## Conflicts of interest

There are no conflicts to declare.

## Data Availability

No primary research results, software or code have been included and no new data were generated or analysed as part of this paper.
